# Swallowing after Oral Oncological Treatment: A Five-Year Prospective Study

**DOI:** 10.3390/cancers15174371

**Published:** 2023-09-01

**Authors:** Caroline M. Speksnijder, Lucía Ortiz-Comino, Anton F. J. de Haan, Carolina Fernández-Lao, Remco de Bree, Matthias A. W. Merkx

**Affiliations:** 1 Department of Oral and Maxillofacial Surgery and Special Dental Care, University Medical Center Utrecht, Utrecht University, 3584 CX Utrecht, The Netherlands; 2 Department of Oral and Maxillofacial Surgery, Radboud University Medical Center, 6525 GA Nijmegen, The Netherlands; 3 Department of Head and Neck Surgical Oncology, University Medical Center Utrecht, Cancer Center, Utrecht University, 3584 CX Utrecht, The Netherlands; 4 Department of Physical Therapy, University of Granada, 18071 Granada, Spain; 5 Department for Health Evidence, Section Biostatistics, Radboud University Medical Center, 6525 GA Nijmegen, The Netherlands; ton.dehaan@radboudumc.nl; 6 Dutch Comprehensive Cancer Centre, 3501 DB Utrecht, The Netherlands

**Keywords:** swallowing, oral function, oral oncology, head and neck cancer

## Abstract

**Simple Summary:**

Swallowing rehabilitation in patients treated for oral cancer is a challenge. Different factors may influence these patients’ swallowing function. Therefore, we aimed to identify factors related to swallowing function up to 5 years after oral cancer treatment. We found that patients who are older at diagnosis, women, and patients who regularly consume alcohol before their treatment may have poorer swallow functioning after oral cancer treatment. Patients that fit these criteria should have their swallowing evaluated during clinical follow-ups and sent to swallowing therapy when needed. During this therapy, optimizing tongue function needs attention to maintain an optimal swallowing function.

**Abstract:**

Background: Swallowing rehabilitation in curative treated patients with oral cancer is still a challenge. Different factors may influence these patients’ swallowing function. The aim of this study was to identify factors associated with swallowing function up to 5 years after cancer treatment. Methods: Swallowing duration and frequency of 5 mL water and 15 mL applesauce were measured in 123 patients treated for oral cancer. Mixed model analyses were performed to identify associated factors. Results: Age influenced all measured swallowing outcomes. Assessment moment, gender, tumor location, maximum tongue force, and tactile sensory function of the tongue were associated with both water and applesauce swallowing duration, tumor classification was associated with water swallowing duration, and alcohol consumption was associated with applesauce swallowing duration. Assessment moment, cancer treatment, maximum tongue force, and tactile sensory function of the tongue were associated with water and applesauce swallowing frequency. Conclusion: Patients who are older at diagnosis, women, and patients who regularly consume alcohol before their treatment may have poorer swallow functioning after curative oral cancer treatment. Patients that fit these criteria should have their swallowing evaluated during clinical follow-ups and sent to swallowing therapy when needed. During this therapy, optimizing tongue function needs attention to maintain an optimal swallowing function.

## 1. Introduction

Head and neck cancer (HNC) involves all neoplasms located in the nasal cavity, paranasal sinuses, oral cavity, salivary glands, pharynx, and larynx. Its worldwide incidence, being the sixth most common cancer, is about 650,000 cases annually. Within HNC, oral cancer reaches 354,864 cases per year, 2% of all global diagnosed cancers [1]. Thanks to curative treatment, the 5-year survival rate of oral cancer ranges from 50 to 92% depending on tumor stage and sublocation [2]. However, due to surgery, radiotherapy, and/or chemotherapy, patients have to deal with many post-treatment complications like swallowing dysfunction [3]. This dysfunction is related to a higher risk of malnutrition, dehydration, and aspiration, which can lead to pneumonia [4]. 

The swallowing mechanism is a centrally controlled process integrated by skeletal support, muscle function, and sensory inputs. During the oral phase of swallowing, the mandible articulates with the skull base, whereas the antero-posterior movement of the tongue pushes the bolus through the oral cavity into the oropharynx. Anteriorly, the lips seal the bolus while the buccal muscles maintain it out of the lateral sulci [5]. For an adequate swallowing process, correct sensory inputs are indeed necessary [6]. 

Nevertheless, after surgery and radiotherapy, impairments such as the inadequate movement of the remaining structures, sensory damage, or damage to the oral mucosa may appear. Then, changes in the positioning of the bolus, labial or buccal muscle strength, oral sensation, or tongue mobility could decrease swallowing function, worsening factors such as duration and frequency [7]. Related to medical treatment, tumor extension into the tongue base, the inadequate functional shaping of the reconstruction, and postoperative radiotherapy have been described as decreasing the swallowing function, consequently decreasing a patient’s quality of life [8,9,10]. Also, age, tumor location and size, and treatment protocol have been reported to impact swallowing [11]. Insight into factors associated with swallowing function like duration and frequency of swallowing in patients treated for oral cancer is of importance to facilitate its management before and after oral oncological treatment. Previous prospective studies have analyzed swallowing function and its associated factors (e.g., age, gender, location of the tumor, treatment protocol) but only up to 2 years after HNC treatment [12,13,14,15,16,17]. It is of importance to know what the swallow function is in long-term survivors, as this can give us as clinicians information to improve swallowing rehabilitation and therefore improve the patient’s quality of life. 

Therefore, the aim of this study was to identify and quantify factors affecting swallowing function over time in patients treated for oral cancer with a 5-year follow-up. Thereby, we compared the swallowing outcomes over time of patients with the swallowing function of healthy persons.

## 2. Materials and Methods

### 2.1. Patients

For this prospective cohort study, the population consisted of patients with a primary malignant tumor in the oral cavity diagnosed at University Medical Center Utrecht (UMCU) or Radboud university medical center (Radboudumc) in Nijmegen, the Netherlands, between January 2007 and August 2009. Patients were included if they were being treated with curative intent using surgery or surgery with adjuvant radiotherapy. Adjuvant radiotherapy was undertaken based on adverse findings from histopathological examination of the surgical specimen. Adjuvant radiotherapy, when given, was administered according to the Dutch Head and Neck Society Guidelines and started within four to six weeks after surgery, with a total dose between 64 and 70 Gy. Patients were excluded from the study if (1) radiotherapy was the primary treatment; (2) they had a second primary tumor; or (3) they were unable to understand Dutch. The protocol was approved by the Ethics Committees of the UMCU and Radboudumc (study ID: NL1200604106) and conducted in accordance with the Declaration of Helsinki. Information regarding the protocol was given to all patients before they gave written informed consent to participate in the study.

Tumor locations included in this study were coded as C00, C02–C06, and C31, as defined by the World Health Organization (WHO)’s International Classification of Diseases for Oncology, third edition [18]. These codes refer to maxillary (upper alveolar process, tuber maxillae, palate, and maxillary sinus: C03.0, C05, and C31.0), mandibular (lower alveolar process, retromolar trigone, buccal mucosa, and lower lips, codes C00.4, C03.1, C06.0, C06.1, and C06.2), and tongue and/or floor-of-the-mouth (TFM) tumors (C02 for tongue and C04 for anterior mouth floor). 

Baseline demographics (age, gender, smoking habit, and alcohol consumption) were registered at the first meeting with the patient. Smoking habit was scored as 0 for non-smokers and infrequent smokers, and 1 for daily smokers. Alcohol consumption was scored as 0 if intake was less than one unit per day on average and 1 if intake exceeded one unit per day on average. Disease data (including tumor location and size by T classification [19]), tumor treatment, resection site, and reconstruction information were extracted from patients’ medical records. 

### 2.2. Standardized Assessment Protocol

Patients were first assessed a maximum of four weeks before their primary treatment (t_0_), at four to six weeks after surgery, and/or four to six weeks after adjuvant radiotherapy (t_1a_ and t_1b_, respectively) six months (t_2_), one year (t_3_), and five years (t_4_) after their primary treatment. Swallowing, maximum tongue force, tongue mobility, the sensory function of the tongue, dental status, and the presence of an obturator prosthesis were assessed at every stage. Healthy persons were assessed once.

### 2.3. Swallowing 

Swallowing was evaluated using 5 mL of water and 15 mL of applesauce. These volumes were chosen to assure the patient’s safety when performing these tests [20]. The duration of swallowing was measured in seconds with a stopwatch from when the cup of water or spoon of applesauce touched the patient’s lip until they indicated that the liquid had been fully swallowed. The swallow frequency was registered by the examiner by making contact placing a finger on the thyroid cartilage level while the patient was swallowing. To improve the statistical analysis of the results by regression models, the frequency outcomes were transformed into binary variables: “one swallow was needed” (0) and “two or more swallows were needed” (1). 

### 2.4. Maximum Tongue Force 

Maximum tongue force was measured in the cranial direction. The device for measuring the tongue force consisted of a strain gauge mounted on a mouthpiece. The strain gauge had a surface area of 110 mm^2^ and a vertical height of 4.5 mm. The strain gauge element was placed between the tongue and the palate at the midline of the tongue 5 mm from the tip. The task of the patient was to press the tongue as hard as possible to the palate. The task was performed twice. The highest tongue force of both efforts was used in this study [21].

### 2.5. Tongue Mobility

To measure tongue mobility, patients were taught to protrude and latero-deviate both sides their tongue as far as possible. Tongue mobility was rated on a three-point ordinal scale: unable to reach the lower lip/mouth corner with the tongue (0); reached the lower lip/mouth corner with the tongue (1); and surpassed the lower lip/mouth corner with the tongue (2). To maintain the clinical applicability of the results, the three variables were recoded into a single variable, selecting the worst functional outcome of the three movements. Results were recoded into a single ordinal variable categorized as no mobility (0); impaired mobility (1); and normal mobility (2).

### 2.6. Sensory Function of the Tongue

Thermal sensory function (thin afferent fibers) and tactile sensory function (thick afferent fibers) were tested by presenting pairs of stimuli: a real stimulus and a sham one. The real and sham stimuli were presented in random order, during two instances of attention that were announced by the examiner while the patient kept their eyes closed. After each pair, the patient had to report the order of real and sham stimulation (forced-choice procedure). Three pairs of stimuli were presented. The magnitude of the test stimulus was chosen as the value at which control subjects could just detect this stimulus with nearly zero errors, so that patients could consistently make the correct choice for uninjured sites. The test sites (right and left) were 10 mm from the tongue tip and 10 mm from the right and left edge of the tongue as much as possible. For analyses, we used the outcome of the (most) affected site. Thermal sensory function was tested using a heat-conducting aluminum rod (diameter 2.0 mm) as a real stimulus (22 °C; touch as well as cold sensory function). The sham stimulus was produced by a non-heat-conducting Perspex rod. Tactile sensory function was evaluated using a Semmes–Weinstein monofilament (Semmes–Weinstein Aesthesiometer, Stoelting Co., Wood Dale, IL, USA) with index number 3.22. The real stimulus was a touch with the filament. The sham stimulus was achieved by approaching the patient with the device while the filament was turned away [21]. The score for reduced thermal or tactile sensory function was 0 and for normal sensory function the score was 1. The sum of thermal sensory function and tactile sensory function outcomes were scored ordinally: unimpaired (0); unilateral impairment (1); and bilateral impairment (2). 

### 2.7. Dental Status

Dental status was organized and assessed into edentulous (0), full denture in upper and lower jaw (1), full denture in upper or lower jaw combined with implant retention in upper or lower jaw (2), full denture with implant retention in upper and lower jaw (3), full denture with implant retention in upper or lower jaw and dentate in the other jaw (4), full denture in upper or lower jaw and dentate in the other jaw (5), and dentate upper and lower jaw (6). Partially dentate jaws were classified as dentate. 

### 2.8. Obturator Prosthesis

When the closure of maxillary defects during surgery was not possible, a temporary obturator based on preoperative assessments and dental casts was made. Approximately one year after surgery, a definitive obturator made of acrylic resin [22] was provided to the patient. The presence or absence of an obturator prosthesis was scored as 1 or 0, respectively.

### 2.9. Statistical Analysis

Normal distribution of variables was checked using the Kolmogorov–Smirnov test. Differences between the baseline characteristics of patients in the different tumor location groups were analyzed with a one-way ANOVA for continuous variables, and a chi-square test or Fisher’s exact test for categorical variables. Differences between mean values were calculated using an independent t-test for continuous variables or a Mann–Whitney U test for ordinal variables and non-normally distributed continuous variables. A *p*-value lower than 0.05 was considered statistically significant.

Patients who received surgery and adjuvant radiotherapy did not show statistically significant differences (*p* < 0.05) between their t_1a_ and t_1b_. Mean values were calculated for maximum tongue force (paired *t*-test), water swallowing frequency (WSF), apple sauce swallowing frequency (ASF), tongue mobility, thermal sensory function of the tongue, tactile sensory function of the tongue, and dental status (Wilcoxon signed-rank test); therefore, only their t_1b_ values were presented.

The distributions of water swallowing duration (WSD) and apple sauce swallowing duration (ASD) were skewed to the right and were therefore logarithmically transformed before the statistical analyses to better fulfill the statistical assumptions. Two linear mixed-effects models with log(WSD) and log(ASD) as outcomes were constructed to assess changes over time and the effects of patient characteristics and clinical parameters (Table 2 and Table 4). To account for within-patient correlations, a random patient factor was added. The assessment from t_0_ to t_4_ and the baseline variables of age, gender, smoking habit, alcohol consumption, tumor location, tumor size, treatment modality, and surgical reconstruction were included as fixed effects in the model. Maximum tongue force (linear), tongue mobility, thermal sensory function of the tongue, tactile sensory function of the tongue, and dental status during the follow-up period were also used as fixed effects. All two-way interactions of the factors within the assessment period were also included in the model to investigate different changes over time for the different variables. 

To build a parsimonious model with a hierarchical structure, factors that were not significant were removed in a backwards fashion, starting with the interactions, meaning that if an interaction was statistically significant, the main effect related to that interaction was also retained in the model. After removal of non-significant interactions, the remaining variables were removed one by one if their contribution was not significant.

For the WSF and ASF, two binary logistic regression models with a random effect for the subject were built (Table 3 and Table 5). First, all variables were included, whereas their interaction with time was added in a forward fashion. Statistically significant interactions were retained in the model. If an interaction was found, then the main effect of the interaction was also retained in the model. Once all interactions were checked for significance, a backwards procedure to remove all non-statistically significant variables was conducted.

Baseline tests and comparisons between patients and healthy persons were executed using SPSS 25.0.0.2 (IBM Corp., Armonk, NY, USA). Mixed models and ordinal logistic regression analyses were performed using SAS 9.4 (SAS Institute, Cary, NC, USA).

## 3. Results

### 3.1. Patient Population

**Figure 1:**
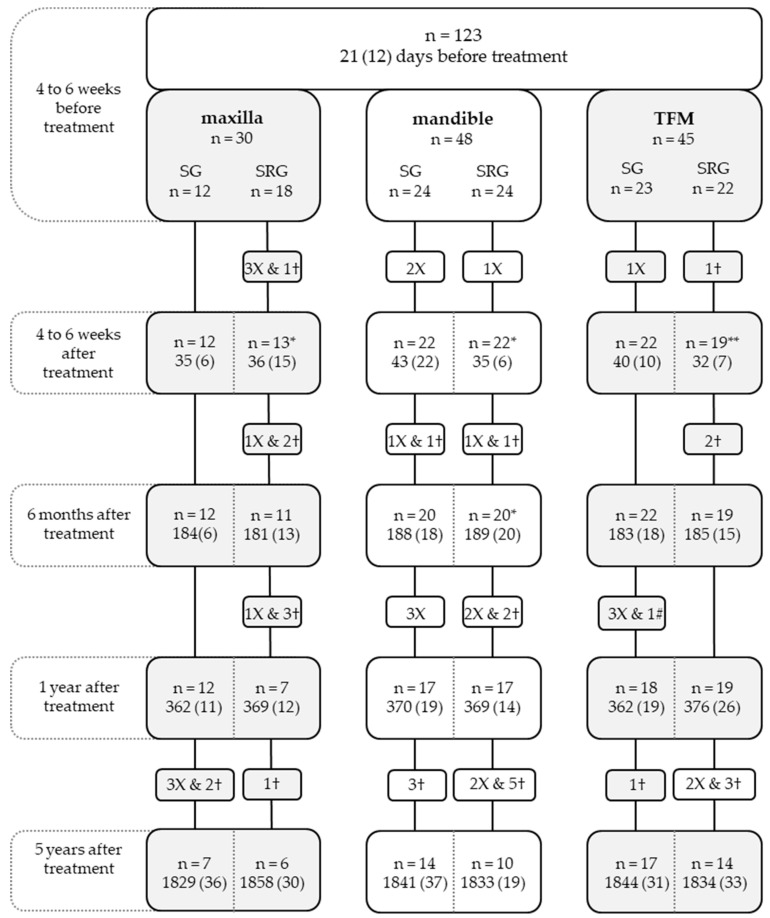
Flow chart showing the number of patients (n) at each assessment and the average time in days (SD) since the primary oncological treatment. TFM, tongue and/or floor of the mouth; SG, surgery group; SRG, surgery–radiotherapy group; RG, radiotherapy group; X, patient(s) stopped participating; †, patient(s) died; *, one missing measurement; **, two missing measurements #, patient excluded because of recurrence of the tumor.

In total, 123 patients were enrolled in this study at t_0_. A total of 30 (24%) with a maxillary tumor, 48 (39%) with a mandible tumor, and 45 with a TFM tumor (37%) were included. After five years, 68 (55%) patients were still involved in the study (Figure 1). Seventeen patients (14%) refused at least one swallowing measurement. At the baseline time point, age, tumor size, surgical reconstruction, ASD, ASF, tongue mobility, and dental status differed significantly between the three tumor location groups (Table 1). The outcomes of maximum tongue force, tongue mobility, and sensory function of the tongue have been published before [23].

WSD, WSF, ASD, and ASF changed significantly over time in the patients with oral cancer (Table 2, Table 3, Table 4 and Table 5). The formulae for WSD, WSF, ASD, and ASF are depicted in Appendix A. However, none of these recovered to the same level as it was before the oral oncological intervention (Table 6). Thereby, differences were found for all comparisons performed between WSD in patients and healthy persons at every measurement moment (*p* < 0.001; Table 6). WSF was significantly different between both groups at t_1_ (*p* = 0.014), t_2_ (*p* = 0.004), t_3_ (*p* = 0.010), and t_4_ (*p* < 0.007). ASD was significantly different between groups at all measurement moments (*p* < 0.001). No statistically significant differences were found for the ASF between groups at any measurement moment (*p* > 0.05).

Comparison of non-estimated WSD and ASD outcomes between measurement moments of patients and outcomes of healthy persons tested by Unpaired *t*-Test. Comparison of non-estimated WSF and ASF outcomes between measurement moments of patients and outcomes of healthy persons tested by Mann–Whitney U Test.

**Table 1:** Demographics, clinical data, and assessments at the baseline categorized by tumor location.

Categorical Variables (n, %)		Maxilla (n = 30)	Mandible (n = 48)	TFM (n = 45)	*p*-Value
Gender	Male	14 (47)	25 (52)	30 (67)	0.179 ^a^
	Female	16 (53)	23 (48)	15 (33)	
Smoking habit	Daily smokers	8 (27)	18 (38)	16 (36)	0.599 ^a^
	Non-smokers and infrequent smokers	22 (73)	30 (62)	29 (64)	
Alcohol consumption	>1 unit alcohol per day	8 (27)	15 (31)	19 (42)	0.328 ^a^
	≤1 unit alcohol per day	22 (73)	33 (69)	26 (58)	
Tumor size by T classification [19]	T1	5 (17)	14 (29)	23 (51)	0.000 ^b^***
	T2	11 (37)	13 (27)	14 (31)	
	T3	1 (3)	3 (6)	4 (9)	
	T4	13 (43)	18 (38)	4 (9)	
Treatment	Surgery	12 (40)	24 (50)	23 (51)	0.600 ^a^
	Surgery and radiotherapy	18 (60)	24 (50)	22 (49)	
Surgical reconstruction	Primary closure	17 (57)	16 (33)	23 (51)	0.000 ^b^***
	Local flap	1 (3)	2 (4)	1 (2)	
	Fasciocutaneous free flap	12 (40)	12 (25)	19 (42)	
	Bone graft/flap	0 (0)	18 (38)	2 (4)	
Water swallowing frequency	Normal	28 (93)	48 (100)	43 (96)	0.177 ^b^
	Impaired swallowing frequency	2 (7)	0 (0)	2 (4)	
Applesauce swallowing frequency	Normal	23 (77)	39 (81)	43 (96)	0.031 ^b^*
	Impaired swallowing frequency	7 (23)	9 (19)	2 (4)	
Tongue mobility	Reaches beyond the lower lip/mouth corner	29 (97)	42 (88)	27 (60)	0.000 ^b^***
	Reaches the lower lip/mouth corner	1 (3)	6 (13)	16 (36)	
	Cannot reach the lower lip/mouth corner	0 (0)	0 (0)	2 (4)	
Thermal sensory function of the tongue	Unimpaired	29 (97)	46 (96)	43 (96)	1.000 ^b^
	Unilateral impairment	1 (3)	2 (4)	2 (4)	
	Bilateral impairment	0 (0)	0 (0)	0 (0)	
Tactile sensory function of the tongue	Unimpaired	29 (97)	44 (94)	40 (89)	0.116 ^b^
	Unilateral impairment	0 (0)	3 (6.4)	5 (11.1)	
	Bilateral impairment	1 (3)	0 (0)	0 (0)	
Dental status	ED	7 (23)	13 (27)	5 (11)	0.000 ^b^***
	FD	7 (23)	8 (17)	13 (29)	
	FD&FDI	0 (0)	2 (4)	4 (9)	
	FD&D	4 (14)	8 (17)	3 (7)	
	FDI&FDI	0 (0)	0 (0)	0 (0)	
	FDI&D	1 (3)	0 (0)	0 (0)	
	D	11 (37)	17 (35)	20 (44)	
**Continuous variables (mean, SD)**					
Age		68.6 (12.3)	66.7 (12.7)	61.4 (13.1)	0.036 ^c^*
Water swallowing duration		2.6 (1.5)	2.9 (1.6)	2.4 (2.2)	0.343 ^c^
Applesauce swallowing duration		3.6 (2.2)	4.4 (3.02)	3.1 (1.7)	0.040 ^c^*
Maximum tongue force		12.9 (6.3)	15.9 (7.7)	15.2 (7.5)	0.651 ^c^

*: *p* < 0.05; ***: *p* < 0.001; ^a^: Chi-square test; ^b^: Fisher’s exact test; ^c^: ANOVA. D: dentate; ED: edentulous; FD: full denture; FDI: full denture on implants; SD: standard deviation; TFM: tongue/floor of mouth.

**Table 2:** The significant coefficients and interactions derived from the mixed model procedure for the water swallowing duration.

	Mixed Model	Main Effects	SE	Interactions with the Assessment Moment									
	Intercept	0.859	0.231										
				Before	SE	After	SE	6 months	SE	1 Year	SE	5 Years	SE
Assessment moment	Before	0.520	0.325										
	After	−0.507	0.201										
	6 Months	−0.294	0.203										
	1 Year	−0.114	0.222										
	5 Years	0	0										
Age		0.009	0.002										
Location	Maxilla	−0.049	0.155	0.220	0.166	0.417	0.174	0.537	0.234	−0.121	0.183	0	0
	Mandible	0.030	0.120	0.314	0.131	0.155	0.138	0.174	0.134	−0.031	0.139	0	0
	TFM	0	0	0	0	0	0	0	0	0	0	0	0
T classification	T1	−0.134	0.150	0.259	0.159	0.023	0.167	0.219	0.176	−0.002	0.180	0	0
	T2	−0.166	0.152	0.243	0.160	0.222	0.170	0.130	0.172	0.099	0.180	0	0
	T3	−0.192	0.207	0.195	0.233	0.010	0.234	0.203	0.229	0.519	0.238	0	0
	T4	0	0	0	0	0	0	0	0	0	0	0	0
Maximum tongue force		−0.009	0.003										
Tongue mobility	Normal mobility	−0.163	0.138	−1.064	0.315	0.081	0.169	−0.004	0.179	0.123	0.192	0	0
	Impaired mobility	−0.083	0.149	−1.006	0.321	0.230	0.179	−0.005	0.186	−0.151	0.197	0	0
	No mobility	0	0	0	0	0	0	0	0	0	0	0	0
Tactile sensory function	Bilateral impairment	0.248	0.214	0.832	0.458	−0.134	0.264	−0.010	0.301	−0.592	0.444	0	0
of the tongue	Unimpaired	−0.134	0.120	0.070	0.194	0.159	0.140	−0.127	0.143	0.129	0.146	0	0
	Unilateral impairment	0	0	0	0	0	0	0	0	0	0	0	0

Coefficients and SE obtained with the mixed model analysis. Main effect of each independent factor is detailed on the “main effects” row. Significant interactions between factors and assessment moment are shown on the “interactions” row. In order to apply these results in practice, coefficients of categorical values should be multiplied by “1” when present and by “0” when absent. Coefficients of continuous variables should be multiplied by the outcome of that factor.

**Table 3:** The significant coefficients and interactions derived from the binary logistic regression for the water swallowing frequency.

	Mixed Model	Main Effects	SE
	Intercept	2.662	1.153
			
Assessment moment	Before	0.930	0.464
	After	0.926	0.458
	6 Months	0.788	0.459
	1 Year	0.306	0.452
	5 Years	0	0
Age		−0.051	0.015
Treatment	Surgery	0.245	0.358
	Surgery and radiotherapy	0	0
Maximum tongue force		0.066	0.024
Tactile sensory function of the tongue	Bilateral impairment	−1.030	0.759
	Unimpaired	1.096	0.356
	Unilateral impairment	0	0

Coefficients and SE obtained with the binary logistic regression analysis. Main effect of each independent factor is detailed on the “main effects” row. Significant interactions between factors and assessment moment are shown on the “interactions” row. In order to apply these results in practice, coefficients of categorical values should be multiplied by “1” when present and by “0” when absent. Coefficients of continuous variables should be multiplied by the outcome of that factor.

**Table 4:** The significant coefficients and interactions derived from the mixed model procedure for the applesauce swallowing duration.

	Mixed Model	Main Effects	SE	Interactions with the Assessment Moment									
	Intercept	1.014	0.200										
				Before	SE	After	SE	6 Months	SE	1 Year	SE	5 Years	SE
Assessment moment	Before	−0.237	0.100										
	After	−0.330	0.103										
	6 Months	−0.196	0.101										
	1 Year	−0.138	0.104										
	5 Years	0	0										
Age		0.014	0.002										
Gender	Male	−0.199	0.072										
	Female	0	0										
Alcohol consumption	≤1 unit alcohol per day	−0.179	0.071										
	>1 unit alcohol per day	0	0										
Location	Maxilla	0.159	0.156	−0.188	0.172	0.420	0.183	0.134	0.179	0.037	0.189	0	0
	Mandible	−0.030	0.131	0.241	0.148	0.346	0.152	0.211	0.151	0.221	0.156	0	0
	TFM	0	0	0	0	0	0	0	0	0	0	0	0
Maximum tongue force		−0.013	0.003										
Tactile sensory function	Bilateral impairment	0.215	0.138										
of the tongue	Unimpaired	−0.219	0.060										
	Unilateral impairment	0	0										

Coefficients and SE obtained with the binary logistic regression analysis. Main effect of each independent factor is detailed on the “main effects” row. Significant interactions between factors and assessment moment are shown on the “interactions” row. In order to apply these results in practice, coefficients of categorical values should be multiplied by “1” when present and by “0” when absent. Coefficients of continuous variables should be multiplied by the outcome of that factor.

**Table 5:** The significant coefficients and interactions derived from the binary logistic regression for the applesauce swallowing frequency.

	Mixed Model	Main Effects	SE	Interactions with the Assessment Moment									
	Intercept	1.806	1.345										
				Before	SE	After	SE	6 Months	SE	1 Year	SE	5 Years	SE
Assessment moment	Before	2.718	0.775										
	After	2.317	0.762										
	6 Months	2.113	0.729										
	1 Year	1.496	0.693										
	5 Years	0	0										
Age		−0.060	0.018										
Treatment	Surgery	2.784	0.886	−3.220	1.074	−3.012	1.074	−2.579	1.050	−2.625	1.028	0	0
	Surgery and radiotherapy	0	0	0	0	0	0	0	0	0	0	0	0
Maximum tongue force		0.081	0.027										
Tactile sensory function	Bilateral impairment	−1.022	0.853										
of the tongue	Unimpaired	1.158	0.403										
	Unilateral impairment	0	0										

Coefficients and SE obtained with the binary logistic regression analysis. Main effect of each independent factor is detailed on the “main effects” row. Significant interactions between factors and assessment moment are shown on the “interactions” row. In order to apply these results in practice, coefficients of categorical values should be multiplied by “1” when present and by “0” when absent. Coefficients of continuous variables should be multiplied by the outcome of that factor.

**Table 6:** Outcomes for the swallowing outcomes at every measurement moment compared with healthy controls.

Patients with Oral Cancer		WSD	WSF	ASD	ASF
t_0_	Mean (SD)	2.68 (1.84)	1.04 (0.24)	3.75 (2.48)	1.18 (0.46)
	Median (IQR)		1 (1–1)		1 (1–1)
t_1_	Mean (SD)	3.22 (2.65)	1.13 (0.41)	5.14 (5.17)	1.42 (0.97)
	Median (IQR)		1 (1–1)		1 (1–1)
t_2_	Mean (SD)	2.94 (1.72)	1.14 (0.38)	4.48 (3.41)	1.32 (0.75)
	Median (IQR)		1 (1–1)		1 (1–1)
t_3_	Mean (SD)	3.08 (1.80)	1.15 (0.47)	4.86 (4.10)	1.40 (0.79)
	Median (IQR)		1 (1–1)		1 (1–1)
t_4_	Mean (SD)	2.99 (1.92)	1.12 (0.32)	4.37 (3.26)	1.37 (0.67)
	Median (IQR)		1 (1–1)		1 (1–1)
Healthy persons	Mean (SD)	1.40 (0.64)	1.00 (0.00)	2.52 (1.07)	1.17 (0.38)
	Median (IQR)		1 (1–1)		1 (1–1)
t_0_ vs. healthy	*p*-value	0.000 ***	0.159	0.000 ***	0.793
t_1_ vs. healthy	*p*-value	0.000 ***	0.014 *	0.000 ***	0.123
t_2_ vs. healthy	*p*-value	0.000 ***	0.004 **	0.000 ***	0.348
t_3_ vs. healthy	*p*-value	0.000 ***	0.010 **	0.000 ***	0.099
t_4_ vs. healthy	*p*-value	0.000 ***	0.007 **	0.000 ***	0.095

*: *p* < 0.05; **: *p* < 0.01; ***: *p* < 0.001. ASD: applesauce swallowing duration; ASF: applesauce swallowing frequency; WSD: water swallowing duration; WSF: water swallowing frequency; t_0_: 4 to 6 weeks before treatment; t_1_: 4 to 6 weeks after treatment; t_2_: 6 months after treatment; t_3_: 1 year after treatment; t_4_: 5 years after treatment.

### 3.2. Water Swallowing Duration

The mixed model analysis showed that the assessment moment (F = 4.18; *p* = 0.002), age (F = 16.67; *p* < 0.001), and maximum tongue force (F = 9.38; *p* = 0.002) significantly influenced WSD (Table 2). Moreover, tumor location (F = 3.81; *p* < 0.001), tumor size (F = 1.8; *p* = 0.047), tongue mobility (F = 3.35; *p* = 0.001), and tactile sensory function of the tongue (F = 2.11; *p* = 0.034) differently affected WSD at each assessment moment. Positive coefficients mean a longer WSD (thus worse performance), whereas negative coefficients mean a shorter WSD (better performance) when all other variables stay the same. 

WSD performance worsened from t_1_ to t_4_. Older patients exhibited poorer swallowing performance (Table 2). The WSD in the group with maxillary tumors worsened from t_0_ to t_1_ and slightly improved from t_1_ to t_4_. In the group with mandibular tumors, WSD increased from t_0_ to t_2_, decreased from t_2_ to t_3_, and increased from t_3_ to t_4_. WSD worsened for the TFM group from t_1_ to t_3_ but improved from t_3_ to t_4_. Tumor classifications lower than pT4 were associated with an improvement in WSD from t_0_ to t_4_, whereas only patients with a pT1 tumor showed an improved WSD performance at t_3_ compared with t_4_. A higher maximum tongue force decreased WSD. The best performance for WSD in patients with a tongue mobility beyond or until the lip was found at t_0_ in comparison to t_1_, t_2_, t_3_, and t_4_. Patients with a bilateral tactile sensory function of the tongue impairment had their worst WSD at t_0_. Patients without tactile sensory function of the tongue impairments had the best WSD at t_4_, although their best performance was reached at t_2_.

### 3.3. Water Swallowing Frequency

The assessment moment (F = 1.88; *p* = 0.11), age (F = 4.47, *p* = 0.035), oncological treatment (F = 4.66; *p* = 0.031), maximum tongue force (F = 8.86; *p* = 0.003), and tactile sensory function of the tongue (F = 3.86; *p* = 0.022) were significant factors for WSF (Table 3). Positive coefficients indicated the probability of a normal frequency when swallowing, whereas the lower negative coefficients indicate that the probability of a normal swallowing frequency is decreasing. WSF worsened from t_0_ to t_4_. Older patients had a higher probability of requiring a greater number of swallows when drinking water. Patients who only underwent surgery performed better than those who underwent surgery and adjuvant radiotherapy. A higher maximum tongue force and the absence of tactile sensory function of the tongue impairments increased the probability of having a normal WSF.

### 3.4. Applesauce Swallowing Duration

The mixed model analysis showed that the assessment moment (F = 3.17; *p* = 0.014), age (F = 33.87; *p* < 0.001), gender (F = 7.63; *p* = 0.006), alcohol consumption (F = 6.23; *p* = 0.014), maximum tongue force (F = 13.65; *p* < 0.001), and tactile sensory function of the tongue (F = 10; *p* < 0.001) significantly influenced ASD, in addition to tumor location (F = 2.58; *p* = 0.009), which influenced ASD differently in every assessment (Table 4). Positive coefficients meant a longer ASD, whereas negative coefficients meant a shorter ASD. 

Patients reached their shortest ASD at t_1_, which then worsened until t_4_. Being older and consuming more than 1 alcohol unit per day worsened ASD. Overall, women performed worse than men. ASD decreased in the patients with maxillary tumors from t_0_ to t_1_, improved until t_3_, and decreased again between t_3_ and t_4_. The patient groups with mandibular and TFM tumors had better ASD than patients in the maxilla group at t_1_ and t_4_. A higher maximum tongue force and the absence of tactile sensory function of the tongue impairments improved ASD.

### 3.5. Applesauce Swallowing Frequency

Age (F = 10.92; *p* = 0.001), maximum tongue force (F = 8.81; *p* = 0.003), and tactile sensory function of the tongue (F = 5.92; *p* = 0.003) were significant factors on the model (Table 5). An interaction between the assessment moment and the curative treatment was found (F = 2.59; *p* = 0.036); therefore, these factors were retained. Positive coefficients indicated the probability of a normal ASF, while negative coefficients indicated that this probability was lower. Older patients had a lower probability of having a normal ASF. Patients treated only with surgery had a better ASF on average and at t_4_ compared with patients treated with surgery and adjuvant radiotherapy. A higher maximum tongue force and the absence of tactile sensory function of the tongue impairments increased the probability of having a normal ASF. 

## 4. Discussion

This prospective cohort study covering 5 years after curative treatment of an oral cavity carcinoma shows how swallowing changes over time in patients treated for oral cancer, and which demographic and clinical factors influence this ability. The best WSD and ASD scores were obtained shortly after the oncological treatment and decreased from this point onwards, while WSF and ASF worsened from t_0_ to t_4_. 

In this study, patient age influenced WSD, WSF, ASD, and ASF; the older the patient, the worse the outcome. In a 2-year prospective cohort study in patients with HNC, this was also found for WSF by using the 100 mL water swallow test [17]. Aging is one of the main risk factors for swallowing dysfunction, as neuromuscular impairments of the swallowing-related structures may occur [8,24,25,26]. Thereby, other aging consequences, such as loss of sensory and cognitive skills, may worsen this function [27]. So, aging adds to swallowing complaints caused by the tumor and its treatment.

Male patients had shorter ASD, corresponding with the results found in another study using a 100 mL water swallowing test (WST) [28]. In contrast, no gender differences were identified for WSD in the present study. These differences may be related to slight differences in the study protocol, as 5 mL of water was used in this study rather than the 100 mL used in the other [28]. Drinking more than 1 unit of alcohol per day before the oncological treatment was associated with a longer ASD. To our knowledge, no information is currently available concerning the relationship between swallowing and alcohol use in patients with HNC. Alcohol can cause damage to the esophageal mucosa, thus worsening swallowing by narrowing the esopahagus through the development of scar tissue [29]. Moreover, chronic alcoholism may cause peripheral neuropathy, resulting in sensory and motor dysfunction [30]. Scar formation on the oral and oropharyngeal mucosa may have increased the ASD in patients who drank more than 1 unit of alcohol per day before treatment. 

Patients with a tumor located on the mandible or maxilla had worse WSD and ASD scores than patients with TFM tumors during the follow-up assessments. Larger tumors (higher T classification) were associated with a worse WSD than the lower T classification. This was also shown in a cross-sectional study, in which a higher T classification was linked to a lower swallowing ability [24]. In the present study, patients in the TFM group had a significantly lower T classification (Table 1), which may have resulted in a better WSD. Moreover, patients with a TFM tumor were younger, while we showed here that older patients generally had worse WSD and ASD scores. In a retrospective study, patients with TFM also presented lower swallowing ability than patients with a mandibular or maxillary tumor in the short term; however, also in this study, the patients had a lower T classification. A TFM tumor, however, is often noticed in an earlier stage than a mandibular or maxillary tumor due to the sensibility of the tongue [31]. Thereby, it is notable that before oral oncological intervention, patients with a mandibular tumor had a worse ASD than patients with a TFM or maxillary tumor (Table 1). The same outcome can also be derived from the linear mixed-effects model analyses in which ASD differs per location group over time and showed the worst ASD in the mandible group before intervention (Table 4). This result shows the importance of mandible functioning during swallowing (semi-)solid food, but it is of less importance during swallowing liquids [32]. However, in this study ASD cannot be explained by the T classification of the tumor in the mandible region, because this factor was not significant (Table 4).

Treatment with surgery and adjuvant radiotherapy was associated with a higher WSF and ASF than surgery alone, which is in accordance with the findings of a previous cross-sectional study on swallowing ability [33] but was not found in a two-year prospective cohort study, which is possibly related to the broader patient group of HNC instead of focusing only on oral cancer [17]. Fibrosis, xerostomia, mucositis, and neuropathy caused by radiotherapy may lead to impaired oral compliance and contractility of swallowing-related musculature [34], affecting WSF and ASF. Indeed, radiation-induced neuropathy on the cranial nerves, and specifically over those related with the swallowing function, implies the worsening of this function, by a decrease in the sensory feedback to the central nervous system [35].

The ASF of patients did not differ from healthy persons at all assessment moments (Table 6). This can be explained by the fact that it is easier to form a bolus of apple sauce (semi-solid food) than to form a bolus of water (liquid food), because more oral motor control is required for a water bolus. So, in patients with oral deficits due to oral cancer and its treatment, it is still possible to form a bolus as in healthy persons [32].

The results of this study show that a greater tongue force improves WSD, WSF, ASD, and ASF. Tongue force and its pressure against the palate are crucial for efficient transport through the oral cavity, and this force is related to the viscosity of the bolus; the higher the viscosity, the greater the force needed [36]. A tongue force decrease is in the literature marked as a consequence of tongue resection [37], which is associated with a higher WSF and ASF [13]. We also found that, before the oncological treatment, unimpaired tongue mobility shortened WSD. The restriction of tongue mobility has generally been related to limited oral functioning [38], as an adequate tongue mobility is required to push the bolus through the oral cavity to achieve a normal swallow [39,40].

This study adds to the known positive effect of a normal tactile sensory function of the tongue on swallowing following oral cancer treatment, as it was associated with better WSD, WSF, ASD, and ASF scores. Specifically, the effects of tactile sensory function of the tongue on WSD differed over time; patients without impairments performed better than those with impairments at 5 years after treatment. Usually, the tactile sensory function of the tongue may be compromised after oral cancer, specifically in those patients requiring free flap reconstruction of the tongue, given the fact that the new flap may not provide the innervation previously given by the tongue resected [41]. Indeed, unimpaired sensory function of the tongue has previously been related to improved masticatory function in patients treated for oral cancer [23], but there is no literature relating a better tactile sensory function of the tongue to better swallowing in this population.

### 4.1. Clinical Implications 

Patients who are older at diagnosis, women, and patients who regularly consumed alcohol before their treatment may have poorer swallow functioning after a curative treatment for oral cancer; thus, patients that fit these criteria should be thoroughly checked for swallowing function during clinical follow-ups (e.g., video fluoroscopy) and sent to swallowing therapy when needed. Patients with larger tumors (higher T classifications) who are treated with surgery and adjuvant radiotherapy are more vulnerable to developing a decreased swallowing function in the years following treatment and therefore at risk for malnutrition. This might be an extra argument for a low-level attitude towards reconstructing the tongue in order to maintain adequate tongue mobility. The rehabilitation of the tongue (mobility, tactile sensory function, and force) is important for optimized swallowing; however, the methodological quality of swallowing exercise studies is low [42,43,44,45], and it is therefore unclear whether these exercises are truly effective. As deficits in swallowing function constitute one of the most common long-term side effects of oral cancer [3,46], taking an individual approach to the patient’s complaints when swallowing is necessary to achieve a better recovery by rehabilitation, not just for swallowing but also for the patient’s quality of life.

### 4.2. Strengths and Limitations

This is to our knowledge the first study to test swallowing using two different liquid consistencies measured over five years after curative oral cancer treatment that also assesses the demographic and clinical factors influencing swallowing over time. Thereby, adding maximum tongue force, tongue mobility, and sensory function of the tongue is unique. The outcomes of the statistical analysis showed the association of these tongue outcomes for swallowing function, which underpins the relevance of our swallowing function measurements. The long follow-up period, prospective study design, and large sample size added to the meticulous data generation and statistical analysis, strengthening our results. 

As HNC encompasses oral cancer, there is a lack of specific literature about the swallow rehabilitation of the oral cancer patient population, and there are no standard methods for evaluating swallowing related to oral cancer treatment. It is therefore difficult to contrast the findings of this study with those of previous research. The methods in this study did not include video endoscopy nor video fluoroscopy, which is the most common technique for evaluating swallowing dysfunction; however, the objective information this system provides regarding structural lesions involving tongue force and sensory function is poor [40]. On the other hand, with our measurements, we could not register information about the presence of oropharyngeal residues after swallowing. Tests like the 100 mL WST [47] or the volume–viscosity swallow test [48] have been proven to be valid tools for the detection of swallowing impairments, but when the present study began, this information had not yet been published. A weakness of this study might be the difference in milliliters to swallow (5 mL) compared to the 100 mL WST used in other studies [28].

Although speech therapy was registered at every measurement moment, the content and frequency of the treatments were unclear. Therefore, the kind of therapy and exercises performed by the patients included in this study were not registered. It can be hypothesized that patients in this study who have had swallowing therapy had a lower WSD, WSF, ASD, and ASF [49]. A mixed methods study showed that patients treated for oral cancer experience, most of all, difficulty in swallowing, chewing, and/or problems with their teeth. To solve these difficulties, these patients prefer a patient-centered rehabilitation program which is based on personal internal and external contextual factors [50].

### 4.3. Future Research

Future research should investigate the relationships between factors affecting swallowing to clarify which groups are more vulnerable to developing a decreased swallowing function. In future research, the influence of different procedures within surgery and radiotherapy must also be investigated in more detail to gain more insight into its influence on swallow function and to further optimize swallowing rehabilitation in patients treated for oral cancer. Therefore, research on the validity and reliability of the used swallowing tests is needed. Studies using a similar analysis to the present research would be beneficial for formulating more robust conclusions. The development of standard evaluations for swallowing will facilitate the use of homogeneous language by specialists and researchers working in the field of oral cancer. Clarifying the main effects related to swallowing impairments will facilitate the development of clinical trials to improve swallowing in patients treated for oral cancer. 

## 5. Conclusions

Patients with and treated for oral cancer have longer swallowing duration compared to healthy persons. The swallowing duration and frequency in these patients are influenced by several factors, up to 5 years after oral cancer treatment. Demographic factors, such as older age, worsen all outcomes, whereas females and alcohol consumers need more time to swallow thicker liquids. Clinical characteristics such as tumor location and size affect WSD, whereas ASD is influenced by tumor location. The frequency of swallows is higher when curative treatment includes surgery and adjuvant radiotherapy. Higher numbers of swallows are only needed in patients when drinking water, not applesauce. Better tongue skills (e.g., force and tactile sensory function) maintain an adequate swallowing function.

## Data Availability

Data can be supplied upon reasonable request from the corresponding author.

## Appendix A. Formulae for the WSD, WSF, ASD, and ASF

Estimated WSD = 0.86 + 0.01Age + 0.03Mand − 0.05Max − 0.13pT1 − 0.16pT2 − 0.19pT3 − 0.01MTF − 0.16TM0 − 0.08TM1 − 0.13TSFt1 + 0.25TSFt3 + t0(0.52 + 0.31Mand + 0.22Max + 0.26pT1 + 0.24pT2 + 0.19pT3 − 1.06TM0 − 1.00TM1 + 0.07TSFt1 + 0.83TSFt3) + t1(−0.5 + 0.15Mand + 0.41Max + 0.02pT1 + 0.22pT2 + 0.01pT3 + 0.08TM0 + 0.23TM1 + 0.16TSFt1 − 0.13TSFt3) + t2(−0.29 + 0.23Mand + 0.54Max + 0.22pT1 + 0.13pT2 + 0.20pT3 − 0.004TM0 − 0.005TM1 − 0.12TSFt1 − 0.01TSFt3) + t3(−0.11 − 0.03Mand − 0.12Max − 0.002pT1 + 0.09pT2 + 0.52pT3 + 0.12TM0 − 0.15TM1 + 0.13TSFt1 − 0.59TSFt3)
Estimated WSF = 2.66 − 0.05Age + 0.93t0 + 0.92t1 + 0.78t2 + 0.30t3 + 0.24Surgery + 0.06MTF − 1.03TSFt1 + 1.09TSFt3
Estimated ASD = 1.01 − 0.20Men + 0.01Age − 0.18No-alcohol − 0.3Mand + 0.16Max − 0.01MTF − 0.22TSFt1 + 0.21TSFt3 + t0(−0.24 + 0.24Mand − 0.19Max) + t1(−0.33 + 0.34Mand + 0.42Max) + t2(−0.19 + 0.21Mand + 0.13Max) + t3(−0.14 + 0.22Mand + 0.38Max)
Estimated ASF = 1.80 − 0.06Age + 2.78Surgery + 0.08MTF − 1.02TSFt1 + 1.15TSFt3 + t0(2.71 − 3.22Surgery) + t1(2.31 − 3.01Surgery) + t2(2.11 − 2.57Surgery) + t3(1.49 − 2.62Surgery)
ASD: Applesauce swallowing duration; ASF: Applesauce swallowing frequency; Mand: Mandible; Max: Maxilla; MTF: Maximum tongue force; No-Alcohol: ≤1 alcohol unit per day; pT1: Tumor stage 1; pT2: tumor stage 2; pT3: tumor stage 3; TSFt1: normal tactile sensory function of the tongue; TSFt3: bilateral impairment on the tactile sensory function of the tongue; TM0: normal mobility of the tongue; TM1: Impaired mobility of the tongue; WSD: Water swallowing duration; WSF: water swallowing frequency
